# Revealing the insoluble metasecretome of lignocellulose-degrading microbial communities

**DOI:** 10.1038/s41598-017-02506-5

**Published:** 2017-05-24

**Authors:** Anna M. Alessi, Susannah M. Bird, Joseph P. Bennett, Nicola C. Oates, Yi Li, Adam A. Dowle, Igor Polikarpov, J Peter W. Young, Simon J. McQueen-Mason, Neil C. Bruce

**Affiliations:** 1 Centre for Novel Agricultural Products, Department of Biology, University of York, York, YO10 5DD UK; 2 Bioscience Technology Facility, Department of Biology, University of York, York, YO10 5DD UK; 3 Grupo de Biotecnologia Molecular, Instituto de Física de São Carlos, Universidade de São Paulo, São Carlos, Brazil; 4 Department of Biology, University of York, York, YO10 5DD UK

## Abstract

Microbial communities metabolize plant biomass using secreted enzymes; however, identifying extracellular proteins tightly bound to insoluble lignocellulose in these microbiomes presents a challenge, as the rigorous extraction required to elute these proteins also lyses the microbes associated with the plant biomass releasing intracellular proteins that contaminate the metasecretome. Here we describe a technique for targeting the extracellular proteome, which was used to compare the metasecretome and meta-surface-proteome of two lignocellulose-degrading communities grown on wheat straw and rice straw. A combination of mass spectrometry-based proteomics coupled with metatranscriptomics enabled the identification of a unique secretome pool from these lignocellulose-degrading communities. This method enabled us to efficiently discriminate the extracellular proteins from the intracellular proteins by improving detection of actively secreted and transmembrane proteins. In addition to the expected carbohydrate active enzymes, our new method reveals a large number of unknown proteins, supporting the notion that there are major gaps in our understanding of how microbial communities degrade lignocellulosic substrates.

## Introduction

Understanding how plant biomass is degraded in soil and compost by mixed microbial communities, has been greatly advanced by the application of ‘omics’ technologies, particularly in determining the way in which the metasecretome allows these communities to interact with one another and their surrounding environment^1–6^. The metasecretome consists of actively secreted extracellular proteins, while the meta-surface-proteome comprises surface-associated proteins either exposed to the microbial surface or intrinsic to the external side of plasma membrane and cell wall^7^. Together the metasecretome and meta-surface-proteome acts as a powerful signature of the processes peculiar to any particular microbial community including recognition, adhesion, transport and communication^8, 9^. While the enzymatic mechanisms of lignocellulose degradation have been characterized in detail in individual microbial species, the microbial communities that efficiently break down plant materials in nature are species-rich and secrete a myriad of enzymes to perform “community-level” metabolism of lignocellulose. Single-species approaches are, therefore, likely to miss functionally important aspects of lignocellulose degradation. However, developing a robust method for metasecretome analysis of lignocellulose-degrading communities in environments such as soil or compost is challenging because many of the proteins involved in plant cell wall degradation are often tightly bound to the biomass^10^. To date, these bound proteins have been difficult to analyze because the stringent conditions needed to extract them generally leads to cell lysis and extensive contamination of the metasecretome with intracellular proteins. Secretomes and exoproteomes have largely been studied in simplified systems using 2D gel-based proteomics on well-characterized and pure-cultured organisms, using very mild extraction protocols and focusing only on soluble proteins retrieved from culture supernatants^11–13^. Although mild washing can prevent lysis of bound microbial cells^14^, this is often not sufficient to liberate tightly adhered proteins^15^.

Here, we report the development of a targeted methodology for metasecretome and meta-surface-proteome extraction and proteomic analysis of compost-derived mixed microbial consortia grown on wheat and rice straw. This methodology, in combination with RNA-seq, led to identification of proteins putatively involved in lignocellulose degradation and nutrient transport from a diverse microbial community.

## Results

### Metasecretome and meta-surface-proteome analysis of microbial consortia from wheat and rice straw compost

**Figure 1:**
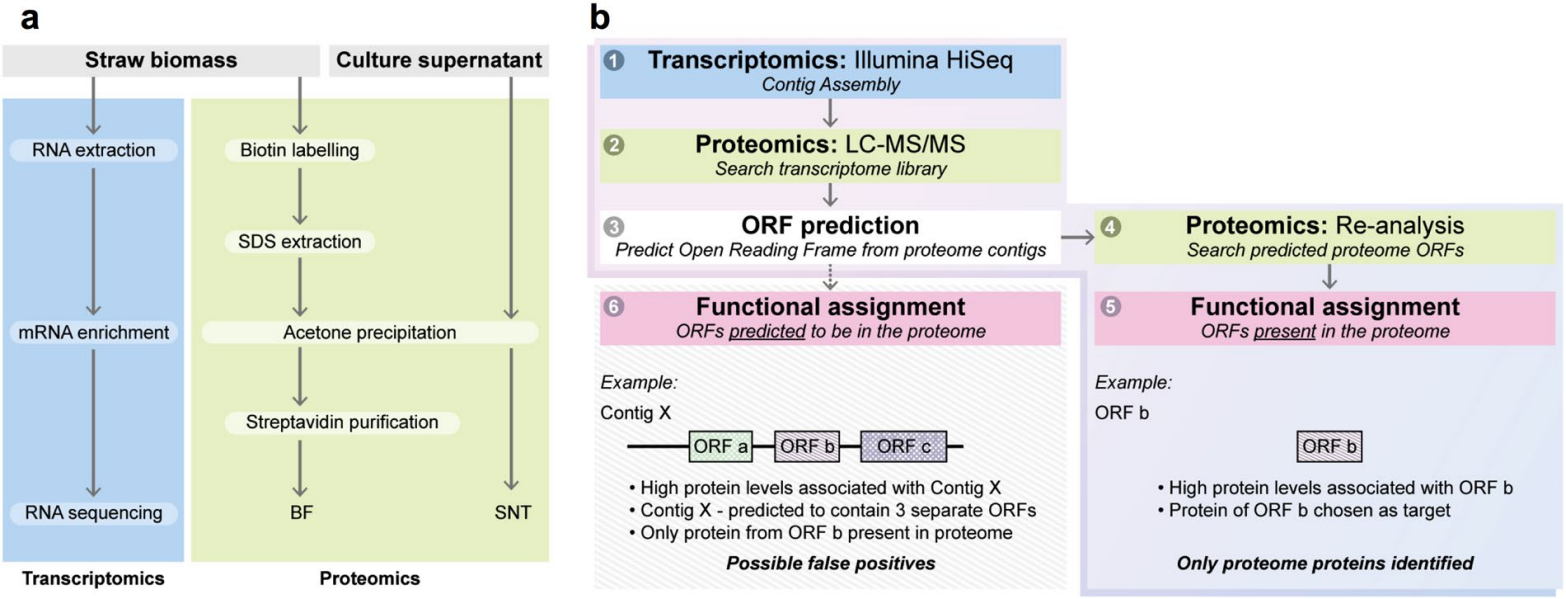
Experimental overview and data analysis of a combined metatranscriptomic and metaproteomic approach to identify unique protein pools in microbial composting communities. (**a**) The experimental overview is split into two sections. For transcriptomic analysis, RNA was extracted from straw biomass and enriched before being sequenced by Illumina HiSeq. For proteomic analysis, soluble protein was precipitated from culture supernatant (SNT), washed and resolublized before analysis, while proteins bound to the straw biomass were first labelled with a sulfo-NHS-SS-biotin tag before solubilizing with a stringent SDS wash (biotin labelled fraction, BF). After precipitation, washing and resolubilization, biotinylated proteins were purified using streptavidin sepharose media. Protein samples were then analysed by LC-MS/MS. (**b**) For data analysis we used the generated metatranscriptomes (1) to identify proteins observed in the various fractions as follows: tandem mass spectra of proteins observed by LC-MS/MS were matched to contigs (2) from the metatranscriptomic analysis, and open reading frame (ORF) predictions were made from these contigs (3). These putative proteomic ORF libraries were used for a second round of analysis of the original LC-MS/MS tandem mass spectra (4). By performing this re-analysis with the putative proteomic ORFs, we only identified proteins that are seen at the protein level (5) and avoided false positives that may have arisen from tandem mass spectral matches to multi-ORF contigs (6).

**Figure 2:**
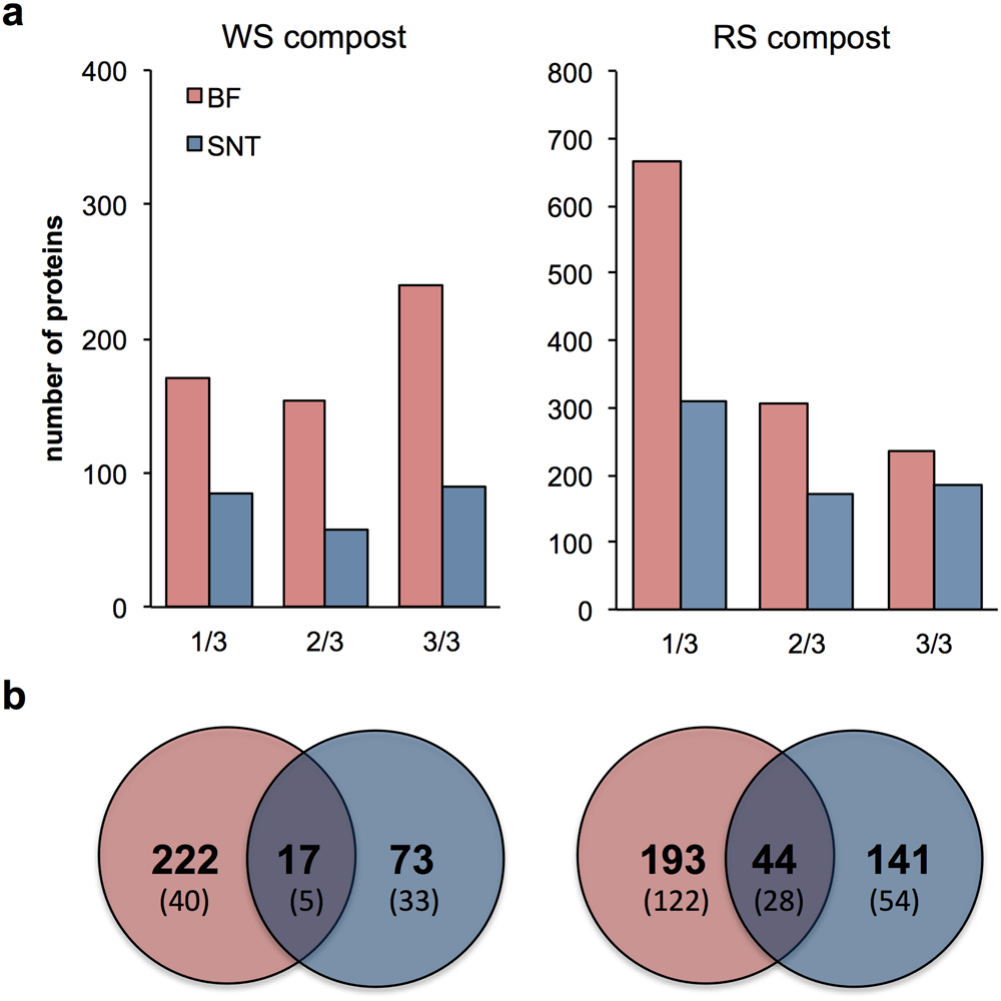
Characterization of the metasecretomes of wheat straw (WS) and rice straw (RS) compost derived communities. (**a**) The number of proteins detected in all three (3/3), two (2/3) or only one (1/3) biological replicate (x-axis) in the biotin-labelled (BF) and supernatant (SNT) fractions. (**b**) Venn plots of unique and shared proteins present in all biological replicates in different designated fractions of WS and RS metasecretomes. In the brackets, numbers of extracellular proteins carrying predicted signal peptide and no transmembrane region targeted by biotinylation or collection of supernatant are shown.

In order to specifically target the extracellular proteins that are tightly bound to the lignocellulosic biomass, we used sulfo-NHS-SS-biotin, which is water soluble but membrane impermeable and non-specifically tags lysine residues and terminal amino groups of proteins. After stringent biomass washing, the biotin-labelled proteins can then be affinity enriched to separate them from the unlabelled intracellular proteins that are released during the washing procedure from the microbes attached to the biomass (Fig. 1). The methodology also proved effective at isolating surface bound and surface exposed integral membrane proteins^16, 17^. We applied our methodology to composting cultures that had been adapted for growth in liquid culture with wheat straw (WS) or rice straw (RS) as the sole carbon sources. In those cultures, the microbial community depends on the presence of exoproteins involved in plant cell wall degradation and nutrient acquisition. During a period of one week, we noted that 19.4 ± 2.1% (s.d.) of WS and 35 ± 0.5% (s.d.) of RS biomass was degraded by the respective composting communities following a substrate weight loss evaluation (see methods). Extracts from the WS and RS cultures were analyzed by LC-MS/MS and searched against metatranscriptomic data generated from the same populations. For the WS communities this resulted in the generation of 4,298 spectra that matched 1,127 unique contigs in the WS metatranscriptomic database, leading to the identification of 723 proteins. The corresponding figures for the RS cultures were 10,996 spectra, 1,757 contigs and 1,624 proteins. Of these proteins, 312 (43.1%) from WS and 378 (23.3%) from RS were present in all three biological replicates and were taken forward for further analysis (Fig. 2a). These proteins, found in the biotin-labelled or supernatant fractions or both, were our samples of the metasecretome and meta-surface-proteome. Based on the MS data, the molar abundance of individual proteins was estimated (Supplementary Tables S1 and S2).

**Figure 3:**
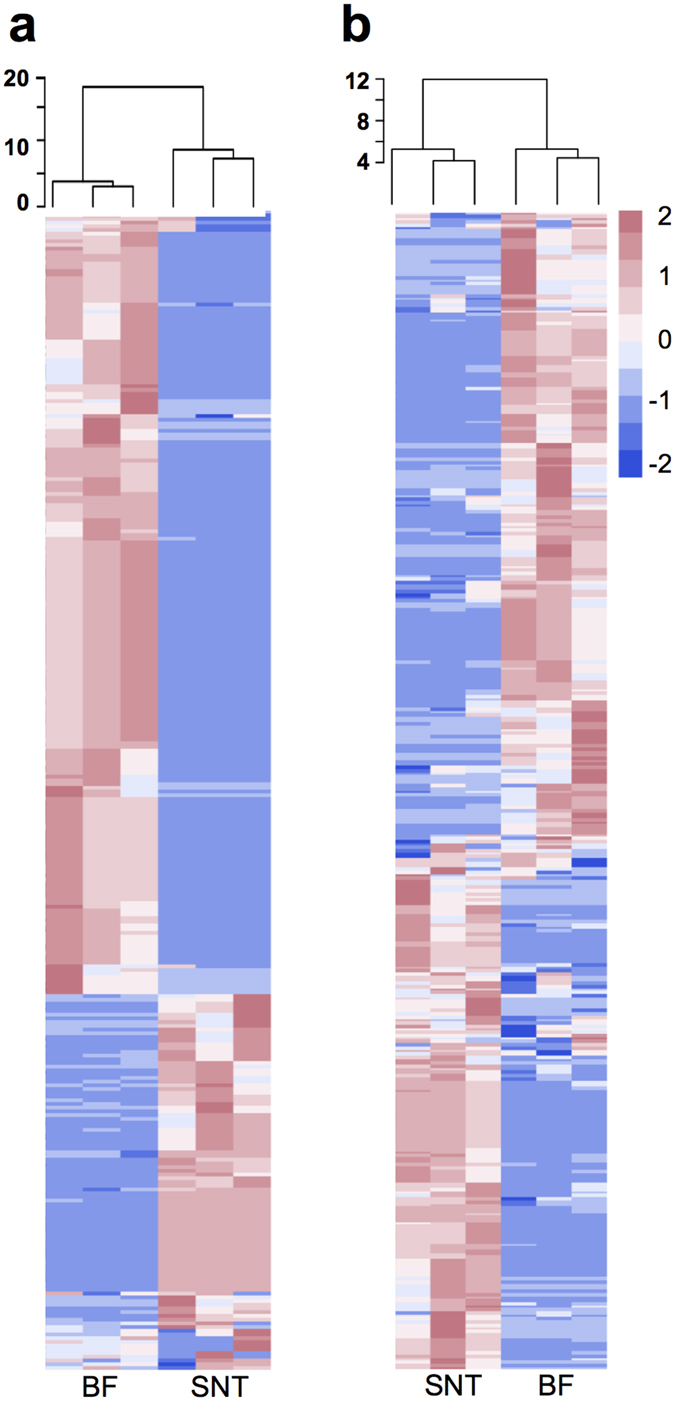
Heatmap representation of the molar abundance for the proteins detected in metasecretome experiment. Heatmaps show molar abundance of proteins that were detected in the biological replicates for (**a**) wheat straw (WS) and (**b**) rice straw (RS) compost communities. Vertical columns represent each biological replicate and the proteome fraction collected for analysis by LC-MS/MS: biotin-labelled (BF) and supernatant (SNT) fractions. Horizontal rows depict proteins identified in the metasecretome. The molar abundance values were centered, scaled in row direction (range from −2 to 2) and used for hierarchical clustering of the samples by using Euclidean distance and average method. Approximately unbiased (AU) *p*-value was calculated via multiscale bootstrap (n = 1000) resampling using pvclust package in R and all the clusters were strongly supported by the data (AU > 0.95). Heatmaps were constructed using pheatmap package in R.

Notably, in the WS samples (n = 312), only 17 of the 239 proteins detected in the biotin-labelled fraction were identified in the culture supernatant, indicating a significant improvement in the detection of specific proteins using our methodology (Fig. 2b). Similarly, the RS samples showed a higher number of unique proteins in the biotin-labelled fraction (n = 193) compared to the culture supernatant (n = 141). The number of proteins present in both fractions was <12% of the total proteins observed (WS = 5.4%, RS = 11.6%) for each of the studied systems (Fig. 2b). Hierarchical clustering analysis revealed dissimilarity between the biotin-labelled fraction and culture supernatant proteomes for both tested microbiomes and demonstrated the reproducibility of the methodology (Fig. 3a,b).

### Phylogenetic analysis of the metasecretomes and meta-surface-proteome and composting cultures

**Figure 4:**
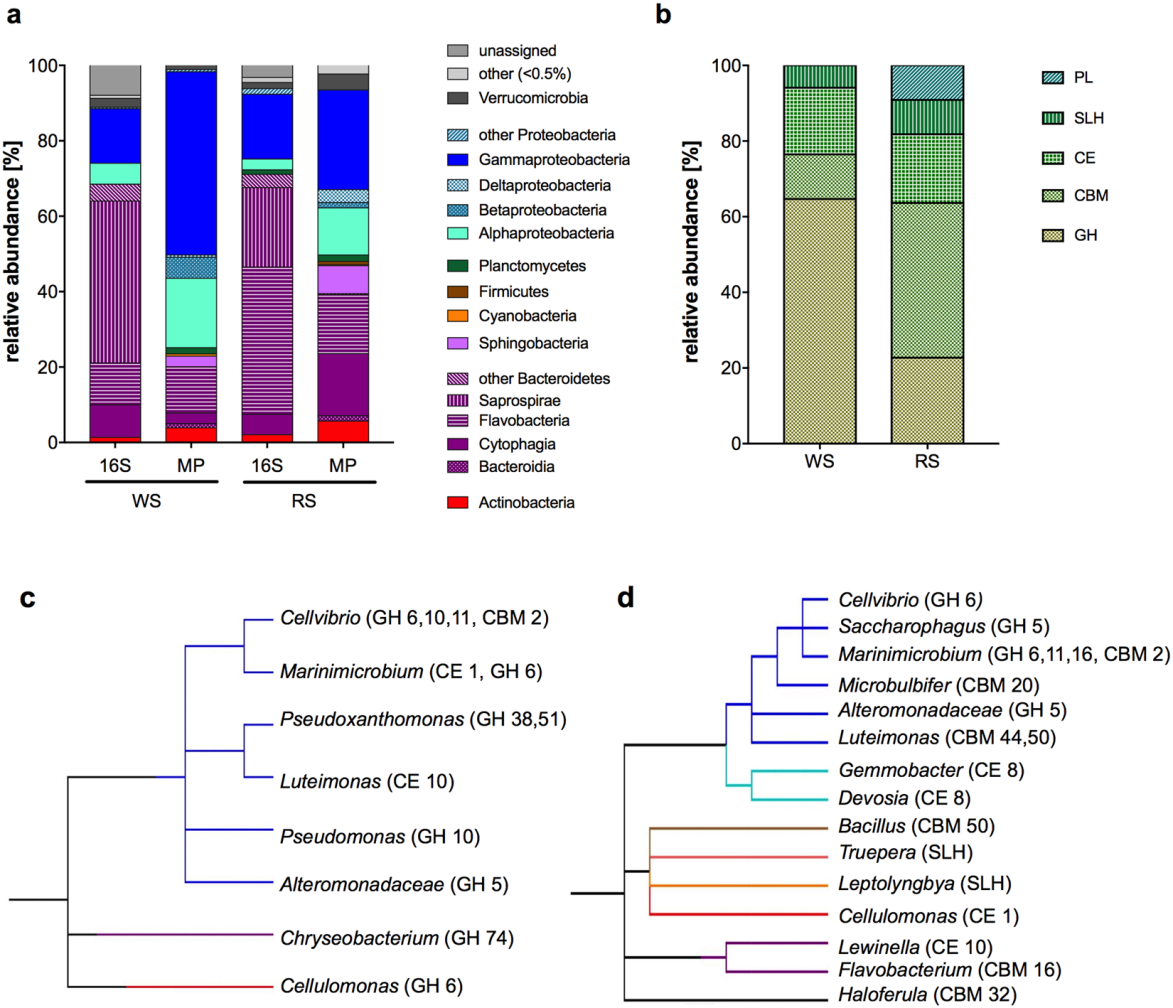
Overview of the metasecretome of wheat straw (WS) and rice straw (RS) microbial composting communities. (**a**) Phylogenetic distribution of bacterial taxa assigned based on 16S rRNA amplicon (16S) sequencing and metasecretome (MP) of WS and RS. (**b**) Distribution of CAZyme proteins (% of total identified CAZymes) in WS and RS compost encoding glycoside hydrolases (GH), carbohydrate binding modules (CBM), carbohydrate esterases (CE), S-layer homology (SLH) modules and polysaccharide lyases (PL). Cladogram displaying genera that contributed to identification of CAZymes in wheat straw (**c**) and rice straw (**d**) metasecretome.

Proteins identified in the supernatant and biotin-labelled fraction datasets were annotated using the basic local alignment search tool (BLASTP) to search against the non-redundant (nr) protein NCBI database, returning 89.1% (n = 279) and 96.0% (n = 363) proteins with a positive hit for the WS and RS datasets, respectively (Supplementary Tables S1 and S2). Phylogenetic assignment of all the proteins identified in the WS and RS cultures was performed based on the BLAST results. Bacterial proteins (WS: n = 179, RS: n = 352) originated mainly from *Proteobacteria* (WS: 73%, RS: 43.7%) and *Bacteroidetes* (WS: 18.9%, RS: 41.2%) phyla (Fig. 4a). Both phylogenetic groups contain members recognized for their role in lignocellulose degradation in compost and were similar in composition to studies reported elsewhere^5, 18^. The WS metasecretome and meta-surface-proteome was dominated by members of *Cellvibrionales* (21%), *Xanthomonadales* (19%) and *Flavobacteriales* (12%), and these classes contributed most of the bacterial proteins identified in both the biotin-labelled and culture supernatant fractions. In addition to the bacterial component of the WS dataset, which accounted for 80 bacterial genera, the majority of 93 eukaryotic proteins were affiliated with peritrich protozoan ciliates of the *Alveolata* group. Analysis of the metasecretome and meta-surface-proteome from the RS cultures indicated that the proteins originated from a more diverse bacterial microbiome than in the WS cultures, comprising 151 bacterial genera classified to multiple classes of *Proteobacteria* (*Cellvibrionales* – 9%, *Xanthomonadales* – 7%, *Rhizobiales* – 6%) and *Bacteroidetes* (*Cytophagales* – 17%, *Flavobacteriales* – 16%) lineages.

To enable a comparison of the phylogenetic results from the metasecretome analysis with the bacterial community profile of the WS and RS cultures, 16S amplicon sequencing was performed. The bacterial microbiome of the WS and RS communities comprised two major taxonomic groups, *Bacteroidetes* (WS: 67.1%, RS: 69.0%) and *Proteobacteria* (WS: 20.4%, RS: 21.5%) (Fig. 4a). Within the *Bacteroidetes* clade the majority of phylotypes in both composting communities were assigned to class *Saprospirae* (WS: 42.9%, RS: 21%), which showed no contribution to the metasecretomes and meta-surface-proteome. In contrast, α- and γ-proteobacteria accounted for the secretion of >50% of the detected proteins in the WS metasecretome and meta-surface-proteome, while the relative abundance of α- and γ-proteobacteria in the composting cultures, based on 16S data, was <20%. The difference in relative abundance between two major phylogenetic groups indicates that the less abundant members of *Proteobacteria* (based on 16S data) were more active contributors to WS and RS metasecretome and meta-surface-proteome than the more abundant *Bacteroidetes*.

### Functional annotation of wheat and rice straw derived metasecretomes and meta-surface-proteomes

From the BLAST annotation it was noted that 43 (13.8%) WS-derived proteins were putative transporters or membrane-bound proteins. Strikingly, amongst those proteins there was a high abundance of TonB-dependent transporters (TBDT) and periplasmic ligand-binding components of ABC (ATPase Binding Cassette) transport systems, which were mainly identified in the biotin-labelled fraction (84%, n = 36). Similarly high percentages of transporters were observed in the RS meta-surface-proteome (19.6%, n = 74).

Following BLASTP searches, we looked for predicted transmembrane helices in proteins identified in the WS and RS meta-surface-proteomes using the TMHMM database (see methods). For the WS meta-surface-proteome, 48 proteins were shown to contain putative transmembrane domains in both the biotin-labelled and culture supernatant fractions. This corresponds to 15.4% of the WS meta-surface-proteome, with distinct proteins sets between the two fractions (biotin: n = 25, 52.1%, supernatant: n = 17, 35.4%). In comparison, 75 proteins in the RS dataset (19.8% of all RS proteins) were predicted to contain transmembrane helices, showing an equal distribution between fractions (biotin: n = 30, 40.0%, supernatant: n = 36, 48.0%). N-terminal signal peptides, required for protein translocation, were predicted to be present in 101 WS and 260 RS proteins (32.3% and 68.8% of the metasecretome, respectively) based on the searches using the SignalP database (see methods, Supplementary Tables S1 and S2). In the RS metasecretome, a high proportion of actively secreted proteins were observed in the biotin-labelled fraction (n = 122, 63%), indicating there had been a significant improvement in targeting extracellular proteins. In contrast, with the WS biotin-labelled metasecretome, there was no observable difference in the proportion of actively secreted extracellular proteins but, importantly, we were able to identify different protein pools by separately screening the supernatant and biotin-labelled fractions.

In order to gain further insight into how the composting communities were degrading lignocellulose, we looked specifically at the distribution of predicted carbohydrate active enzymes (CAZymes, Supplementary Tables S1 and S2). We found that 5.45% (17/312) and 5.8% (22/378) of proteins were assigned to CAZy proteins in the WS and RS samples, respectively. In both compost-derived communities the majority of CAZy proteins were located in the biotin-labelled fraction (WS: n = 14, 82.3%, RS: n = 15, 68.2%). The molar abundance of CAZy-annotated proteins in the WS metasecretome reached 2.3% in the biotin-labelled fraction and 1.2% in the culture supernatant, whereas in RS metasecretome CAZy-assigned proteins accounted for 5.9% in the biotin-labelled fraction and 0.8% in the culture supernatant. We note that despite differences in carbon source composition and inocula, both composting communities display similar numbers and distribution of CAZymes (Fig. 4b). The diversity of microorganisms producing CAZymes was higher in RS cultures, though both systems showed the presence and contribution of CAZymes from well-known lignocellulolytic bacteria such as *Cellulomonas* and *Cellvibrio* ^19^ (Fig. 4c,d). An array of hydrolytic GH5 and GH6 enzymes^20^ involved in endo- and exo-hydrolysis of cellulose chains was identified in the WS metasecretome. In addition, a number of hemicellulose degrading enzymes from GH10, GH11 and GH51 families were identified in the WS system. The RS metasecretome displayed the presence of xylanases (GH11) and cellulases (GH5, GH6) but the most abundant proteins were assigned to various families of carbohydrate binding modules^20^ (CBM 16, 20, 32, 44, 50). Those proteins were often annotated as hypothetical proteins displaying a low level of sequence similarity to previously characterized proteins. Both compost-derived metasecretomes lacked potential ligninases e.g. laccases, lignin peroxidases and also lytic polysaccharide monooxygenases (LPMOs), which are classified as proteins with auxiliary activities (AA) in the CAZy database.

**Figure 5:**
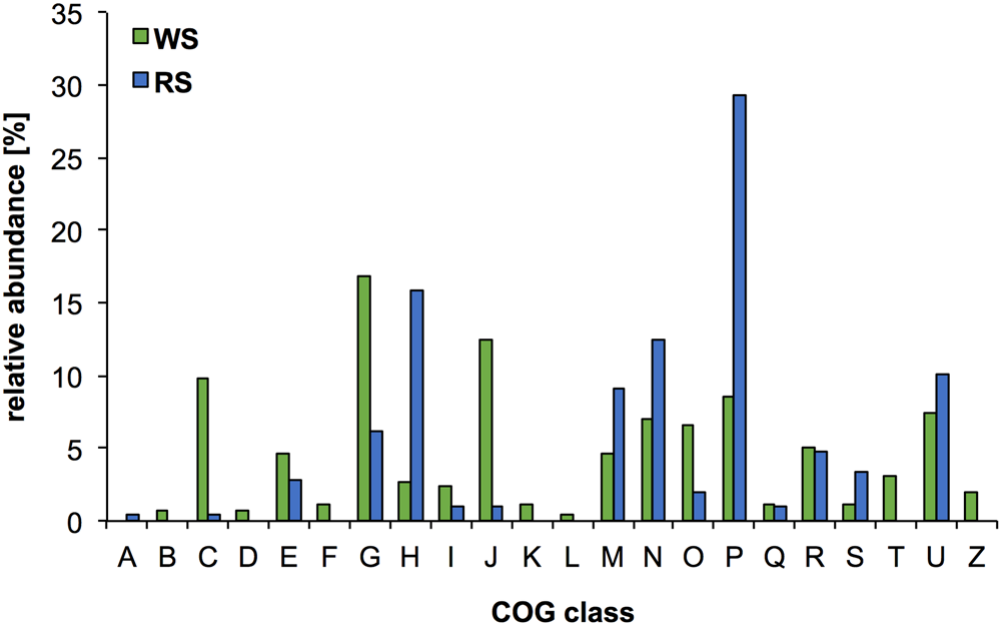
Comparison of clusters of orthologous groups (COGs) in metasecretome of wheat straw (WS) and rice straw (RS) compost derived communities. The predicted proteins identified in metasecretome were mapped to different COGs using WebMGA server and RPSBLAST program. [A] RNA processing and modification [B] Chromatin structure and dynamics [C] Energy production and conversion [D] Cell cycle control, cell division, chromosome partitioning [E] Amino acid transport and metabolism [F] Nucleotide transport and metabolism [G] Carbohydrate transport and metabolism [H] Coenzyme transport and metabolism [I] Lipid transport and metabolism [J] Translation, ribosomal structure and biogenesis [K] Transcription [L] Replication, recombination and repair [M] Cell wall/membrane/envelope biogenesis [N] Cell motility [O] Post-translational modification, protein turnover, and chaperones [P] Inorganic ion transport and metabolism [Q] Secondary metabolites biosynthesis, transport, and catabolism [R] General function prediction only [S] Function unknown [T] Signal transduction mechanisms [U] Intracellular trafficking, secretion, and vesicular transport [Z] Cytoskeleton.

Following functional classification using the cluster of orthologous groups (COGs) protein database, we assigned 256 and 208 functions for 67% (n = 209) and 46% (n = 174) of predicted proteins in the WS and RS metasecretomes, respectively (Fig. 5). Proteins involved in carbohydrate metabolism and transport dominated the WS cultures (n = 43, 16%). Those proteins were related to functions dealing with lignocellulose degradation and sugar translocation including the periplasmic component of the ABC-type transport system. The second most abundant cluster was involved in translation, ribosomal structure and biogenesis indicating presence of intracellular proteins in the WS metasecretome. The RS metasecretome was enriched in proteins involved in transport of inorganic ions. The majority of the 61 proteins classified to this cluster showed homology to outer membrane receptor proteins for Fe, ferrienterochelin and colicin transport. The high abundance (n = 33, 15%) of proteins involved in cobalamin transport and its metabolism was also more dominant in the RS cultures.

In addition, we also identified a high percentage of uncharacterized and unknown proteins in both metasecretomes: 37% of all WS proteins had matches to hypothetical/predicted proteins in the nr-database, while almost half (49%) of all observed RS proteins matched hypothetical/predicted proteins, with 13% having no hits at all using the selected threshold (Supplementary Tables S1 and S2).

## Discussion

In this paper, we describe a methodology, which has allowed an unprecedented depth of analysis of the proteins present in the metaesecretomes of lignocellulose-degrading mixed microbial communities derived from wheat straw and rice straw compost, respectively. As previously reported, identification of proteins by tandem mass spectroscopy requires a reference database, often only available for model microorganisms^21^. Hence, we screened the tandem mass spectrometry data against the transcriptomics database obtained by RNA-Seq from the respective cultures used in this study.

In compost the functional diversity is driven by multiple environmental factors including source of plant material, soil residues, water and oxygen content and seasonal temperature^22, 23^. The composting communities are, therefore, dependent on the presence of a diverse range of actively secreted extracellular proteins involved in plant cell wall degradation and cell-associated transport proteins for rapid nutrient uptake^5, 24^. Many of those proteins remain tightly bound to the substrate by specialized carbohydrate-binding domains^10^. As hypothesized, our targeted proteomics provided a detailed picture of the metasecretome and the dynamics of the composting microbial communities acting on the insoluble substrates provided by rice and wheat straw. In agreement to previous studies^1, 24, 25^, a diverse group of CAZymes was identified in the compost samples that are required to degrade the component parts of lignocellulose. Although, the proportion of predicted CAZymes in our metasecretomes, is only around 5%, this is similar to other reports that applied carbon enrichment^14, 26^. This reflects the abundance of CAZyme hits in the metagenomics data from lignocellulose degrading microbiomes in which some GH families (e.g. GH3, GH43) are shown to be more prevalent (10 hits per million reads), whereas other GH families (e.g. GH5, GH11) are less abundant within metagenome assemblies (<1 hit per million reads)^24^. In contrast to other studies^26, 27^, we have not detected proteins affiliated with the AA class of CAZymes. Those proteins play important roles in oxidative degradation of polysaccharides and lignin^28^. Many of the AA identified proteins are produced by fungi^29^ and, since both composting communities displayed no proteins affiliated with this kingdom, the lack of fungal AAs is not surprising. The bacterial AAs might have slipped detection possibly due to their low abundance and/or the stringent method applied for data analysis in this study. However, our study showed the presence of proteins involved in cellulose degradation such as cellobiohydrolases (GH6) and endoglucanases (GH5, GH9) which were not reported in previous studies^26, 27^.

A variety of transporters and membrane proteins (such as OmpA/MotB-containing proteins) were identified in both meta-surface-proteomes. This implies that a considerable number of proteins are involved in the uptake of a diverse range of compounds generated from the degradation of lignocellulose and reflects the different nutritional requirements of the microbial consortia^14^.

The majority of identified proteins were assigned to *Proteobacteria* and *Bacteroidetes* lineages. Both phyla contain members recognized for their role in lignocellulose degradation in compost and were similar in composition to studies reported elsewhere^5, 18^. Compost microbiomes comprise taxa from various phylogenetic backgrounds including bacteria, fungi and other eukaryotes^6, 18^. We observed that the majority of proteins identified in the WS and RS metasecretome originated from bacteria. As previously reported, soil and compost ecosystems contain a high diversity of protists, which play important role in controlling bacterial turnover and community composition, recycling of nutrients and promotion of plant growth^30^. We have observed a high proportion of protozoan proteins in the WS system but not in RS cultures. We also noted that a significant proportion of the intracellular proteins identified in the WS cultures were produced by the protists. This explains a lower proportion of proteins with signal peptides in the WS cultures than in the RS cultures. Further, fungal proteins were not detected in the compost-derived cultures indicating low abundance of fungal taxa, which possibly reflects the liquid shake flask culturing conditions that were most likely more favourable for bacterial growth.

We also found that, in contrast to the WS system, the RS cultures contained significantly more proteins annotated as hypothetical/unknown or for which no BLAST hits were found when searched against the non-redundant protein database. Many of these proteins contain CBMs^31^ but no catalytic domains identifiable from previously characterized proteins, suggesting that much of the metasecretome is yet to be understood.

In summary, we have successfully adapted the use of sulfo-NHS-SS-biotin to target extracellular proteins from complex composting communities. This methodology in combination with transcriptomics led to the identification of a significantly higher number of unique proteins compared to collecting soluble proteins from the culture supernatant alone. To the best of our knowledge, this has provided the most sensitive and reproducible method developed thus far to characterize complex metasecretomes. This strategy made it possible to identify many proteins putatively involved in lignocellulose degradation and nutrient transport. The identification of large numbers of uncharacterized proteins offers an invaluable opportunity to expand our knowledge of lignocellulose degradation, with the potential to mine for new commercially valuable biomass processing enzymes. In addition, this protein-labelling approach could be applied to a variety of complex microbial ecosystems to provide details on major metabolic players and the function and contribution of the metasecretome in those communities.

## Materials and Methods

### Wheat and rice straw composting cultures

The cultures used wheat or rice straw enriched compost as an inoculum, which was mixed and homogenized before inoculating at 1% (w/v) into minimal medium (KCl 0.52 g/L, KH_2_PO_4_ 0.815 g/L, K_2_HPO_4_ 1.045 g/L, MgSO_4_ 1.35 g/L, NaNO_3_ 1.75 g/L, Hutner’s trace elements)^32^ containing 5% (w/v) wheat straw or 2.5% (w/v) rice straw as a sole carbon source. The cultures were grown at 30 °C with 150 rpm agitation for 1 week before nucleic acids and proteins were harvested. Both wheat straw and rice straw cultures were prepared in three biological replicates. The residual WS or RS biomass was harvested by centrifugation (4,500 × g, 10 minutes), dried (50 °C oven) and weighted. Biomass from three biological replicates were weighted and compared to control cultures without the compost inoculum.

### Metatranscriptomics and 16S amplicon sequencing

#### Nucleic acids extraction

DNA/RNA was extracted from the cultures using an adapted Griffiths protocol^33^ to a microcentrifuge tube containing 0.5 g acid-washed zirconia beads. Equal volumes of CTAB buffer (10% CTAB in 0.7 M NaCl, 240 mM potassium phosphate buffer, pH 8.0) and phenol:chloroform:isoamyl alcohol (25:24:1, pH 8.0) were added and after mixing the samples were disrupted in a TissueLyser II (Qiagen) for 2.5 min at speed 28 s^−1^. The aqueous phase was extracted with 1 volume of chloroform:isoamyl alcohol (24:1). The nucleic acids were precipitated by adding 2 volumes of 1.6 M NaCl/20% PEG8000 buffer (0.1% DEPC treated) during overnight incubation at 4 °C. The resulting pellet was washed twice with ice cold 70% ethanol and resuspended in RNase/Dnase-free water.

#### RNA-seq and data assembly

Prior to sequencing, total RNA samples were treated with RTS DNase (MoBio) according to the manufacturer’s instructions, followed by elimination of small RNAs and purification using a Zymo Research clean up and concentrator kit. Ribosomal RNA was removed from a 2.5 µg aliquot of total RNA (using an Epicentre Epidemiology kit) to obtain an mRNA-enriched sample. The profile of ribosomal-depleted samples was assessed using an Agilent Bioanalyzer mRNA analysis kit. The cDNA libraries were constructed using 100 ng of ribosomal-depleted RNA and the adapted TruSeq RNA v2 protocol (Illumina 15026495 Rev.B). The constructed libraries were normalized using elution buffer (Qiagen) and pooled in equimolar amounts into one final 12 nM pool. The libraries were diluted further to a final concentration of 10 pM and were spiked with 1% PhiX before loading onto the Illumina cBotTemplate. Hybridization and first extension were carried out on the cBot utilizing the TruSeq Rapid PE Cluster Kit v1 prior to the flow cell being transferred onto the Illumina HiSeq2500 (RS: HiSeq3000) for the remainder of the clustering process performed following the manufacturer’s instructions. The sequencing chemistry was TruSeq Rapid SBS Kit v1 using HiSeq Control Software 2.2 and RTA 1.18. The library pool was run in a single lane for 100 cycles of each paired-end read. Reads in bcl format were demultiplexed based on the 6 bp Illumina index by CASAVA 1.8, allowing for a one base-pair mismatch per library, and converted to FASTQ format by bcl2fastq. The sequenced libraries were searched against Silva_115 database^34^ to identify ribosomal RNA genes using Bowtie2 software^35^. Those reads as well as orphans and poor quality sequences were removed with the ngsShoRT software and the remaining reads were pooled prior to assembly with *de novo* Trinity package^36^.

#### 16S amplicon sequencing

Small subunit (SSU) rRNA gene sequences were amplified using primer pairs covering the bacterial V4 (forward F515: 5′-GTGCCAGCMGCCGCGGTAA-3′, reverse R806: 5′- GGACTACHVGGGTWTCTAAT-3′) region^37^. The reactions for amplicons were carried out using Phusion High-Fidelity DNA Polymerase (Finnzymes OY, Espoo, Finland). The amplified fragments were purified with Agencourt AMPure XP (Beckman Coulter). The quantity and quality of the purified PCR products were analysed using an Agilent Tape Station with an Agilent DNA 1000 kit. Amplicons were barcoded using an Nextera XT Index kit. The libraries were quantified using Invitrogen Qubit, diluted to 4 nM and an equal amount from each library with unique indices was pooled to create the final library. The library was denatured and spiked with PhiX control to a final concentration of 30% (v/v). The libraries were sequenced on a MiSeq system using v3 reagents (300-cycles).

#### Data analysis using QIIME pipeline

Demuliplexed FastQ files were quality filtered using the split_library.py script^38^. Chimeric sequences were removed using usearch61 and the remaining nonchimeric sequences were clustered by pick_open_reference_otus.py into OTUs (Operational Taxonomic Units) at 97% similarity using UCLUST as the clustering method^39^. The bacterial OTUs were taxonomically annotated using the Greengenes (gg_13_8, March, 2015) database^40^. Biom-formatted OTU tables were created and filtered to exclude OTUs containing fewer than ten sequences.

### Metasecretome and meta-surface-proteome extraction and analysis

#### Sample preparation

Soluble supernatant protein extraction (supernatant protein - SNT) used clarified culture supernatant from straw cultures that was passed through 0.22 µm PES filter units. Soluble proteins were precipitated with 5 volumes of 100% (v/v) ice-cold acetone overnight at −20 °C. The resulting protein pellets were washed twice with 80% ice-cold acetone, air-dried and resuspended in 0.5x PBS (68 mM NaCl, 1.34 mM KCl, 5 mM Na_2_HPO_4_, 0.88 mM KH_2_PO_4_) buffer.

To extract proteins bound to the straw biomass (bound fraction protein - BF), two grams of straw biomass was washed twice with ice-cold 0.5 × PBS and resuspended in 0.5 × PBS supplemented with 10 mM EZ-link-Sulfo-NHS-SS-biotin (Thermo Scientific). Samples were mixed thoroughly for 1 hour at 4 °C. The reaction was quenched at 4 °C for 30 min by the addition of 50 mM Tris-HCl, pH 8.0. Biotin residues were removed by washing biomass twice with ice-cold 0.5 × PBS. The proteins were extracted with pre-warmed SDS (2% w/v, 60 °C) and samples were mixed at room temperature for 1 hour. The mixture was centrifuged and proteins were precipitated as described above. BF protein pellets were solubilized in 1 × PBS (137 mM NaCl, 2.7 mM KCl, 10 mM Na_2_HPO_4_, 1.8 mM KH_2_PO_4_) containing 0.1% SDS, and passed through a 0.22 µm PES filter unit before being loaded onto pre-washed (0.1% SDS in 1x PBS buffer) streptavidin columns (Thermo Scientific). Proteins were incubated on the columns 1 hour at 4 °C to aid binding, before being washed with 0.1% SDS in 1x PBS. Columns were incubated overnight at 4 °C with elution buffer of 50 mM DTT in 1 × PBS. Sequential elution of proteins from the streptavidin column was done by loading 4 times 1 mL 50 mM DTT in 1 × PBS, collecting the fraction and incubating the column for 1 hour before next elution. Eluted fractions were freeze-dried, resuspended in 2 mL distilled water and desalted (Zeba, 7 K MWCO, Thermo Scientific). SNT and BF protein samples were subjected to SDS-PAGE on 4–12% Bis-Tris gels, and protein bands were excised and cut into 1 mm pieces which were stored at −80 °C prior to analysis.

#### Protein In-Gel Digestion

Gel slices were washed twice with 50% (v/v) aqueous acetonitrile containing 25 mM ammonium bicarbonate, reduced and alkylated with 10 mM DTE, and *S*-carbamidomethylated with 50 mM iodoacetamide. Following dehydration with acetonitrile, gel pieces were digested with the addition of 0.2 µg sequencing-grade, modified porcine trypsin (Promega) in 25 mM ammonium bicarbonate and incubated at 37 °C overnight. Peptides were extracted from the gel by washing three times with 50% (v/v) aqueous acetonitrile, before drying down in a vacuum concentrator and reconstituting in 0.1% (v/v) aqueous trifluoroacetic acid.

#### Liquid Chromatography Tandem MS

Samples were loaded onto a nanoAcquity UPLC system (Waters) equipped with a nanoAcquity Symmetry C_18_, 5 µm trap (180 µm × 20 mm Waters) and a nanoAcquity HSS T3 1.8 µm C_18_ capillary column (75 μm × 250 mm, Waters). The trap wash solvent was 0.1% (v/v) aqueous formic acid and the trapping flow rate was 10 µl min^−1^. The trap was washed for 5 min before switching flow to the capillary column. The separation used a gradient elution of two solvents (solvent A: 0.1% (v/v) aqueous formic acid; solvent B: acetonitrile containing 0.1% (v/v) formic acid): linear 2–30% B over 125 min then linear 30–50% B over 5 min. The flow rate for the capillary column was 300 nL min^−1^ and the column temperature was 60 °C. All runs then proceeded to wash with 95% solvent B for 2.5 min. The column was returned to initial conditions and re-equilibrated for 25 min before subsequent injections.

The nanoLC system was interfaced with a maXis HD LC-MS/MS System (Bruker Daltonics) with a CaptiveSpray ionization source (Bruker Daltonics). Positive ESI- MS & MS/MS spectra were acquired using AutoMSMS mode. Instrument control, data acquisition and processing were performed using Compass 1.7 software (microTOF control, Hystar and DataAnalysis, Bruker Daltonics). Instrument settings were: ion spray voltage: 1,450 V; dry gas: 3 L min^−1^; dry gas temperature 150 °C; collision RF: 1,400 Vpp; transfer time: 120 ms; ion acquisition range: *m/z* 150–2,000. AutoMSMS settings specified: absolute threshold 200 counts, preferred charge states: 2–4, singly charged ions excluded. Cycle time and spectra rates were adjusted for individual samples as follows: WS, cycle time: 3 s, MS spectra rate: 2 Hz, MS/MS spectra rate: 2 Hz at 2,500 cts increasing to 12 Hz at 250,000 cts or above; RS, cycle time: 1 s, MS spectra rate: 5 Hz, MS/MS spectra rate: 5 Hz at 2,500 cts increasing to 20 Hz at 250,000 cts or above. Collision energy and isolation width settings were automatically calculated using the AutoMSMS fragmentation table. A single MS/MS spectrum was acquired for each precursor and former target ions were excluded for 0.8 min unless the precursor intensity increased fourfold.

### Data analysis

Tandem mass spectral data were searched against either the wheat straw or rice straw compost metatranscriptomes (see corresponding accession number in the European Nucleotide Archive; WS: PRJEB12382, RS: PRJEB12448) using a locally-run copy of the Mascot program (Matrix Science Ltd., version 2.4), through the Bruker ProteinScape interface (version 2.1). Search criteria specified: Enzyme, Trypsin; Fixed modifications, Carbamidomethyl (C); Variable modifications, Oxidation (M) and Deamidation (NQ); Peptide tolerance, 10 ppm; MS/MS tolerance, 0.1 Da; Instrument, ESI-QUAD-TOF.

Nucleotide sequences for contigs identified by Mascot as having matches to observed proteins were retrieved from the metatranscriptomic databases using Blast-2.2.30 + Standalone^41^. EMBOSS application getorf^42^ was used to generate all possible open reading frames (ORFs) from these matched contigs, defined as any region >300 bases between a methionine start (ATG) and STOP codon. These ORF libraries were converted into amino acid sequences and then used as the databases for a second round of searches with the original tandem mass spectral data. Results were filtered through ‘Mascot Percolator’ and adjusted to accept only peptides with an expect score of 0.05 or lower. An estimation of relative protein abundance was performed as described by Ishihama^43^. Molar percentage values were calculated by normalising the Mascot derived emPAI values against the sum of all emPAI values for each sample.

Protein sequences from ORFs identified as being present in the metaesecretomes were annotated using BLASTP searching against the non-redundant NCBI database with an E-value threshold of 1 × 10^−20^. Additionally, protein sequences were annotated using dbCAN^44^ to identify likely carbohydrate active domains (if alignment length >80 aa, E-value < 1 × 10^−5^ was used, otherwise E-value < 1 × 10^−3^ was applied). Subcellular localization was predicted using TMHMM v. 2.0^45^ server. SignalP v. 4.1^46^ database was used to identify signal peptides from Eukaryotes, Gram-negative and Gram-positive bacteria with default cut-off values. Heatmaps were constructed using package pheatmap v. 1.0.8 in R.

## Electronic supplementary material

Supplementary Table S1

Supplementary Table S2
